# Extra-Limites

**Published:** 1845-10

**Authors:** 


### EXTRA-LIMITES.

### TESTIMONIAL TO J. R. MARTIN, Esq.

To Colonel James Young, Dr. A. R. Jackson,
and John Turner, Esq. Surgeon.

Gentlemen,

Enclosed I have the honor to hand to you a Bill, drawn by the Union Bank Secretary upon their London correspondents at ten months date, for the sum of £250. payable to Mr. J. R. Martin, or order.

If you will take the trouble to peruse the accompanying extracts from the public papers here of dates 31st July and 13th November last, you will understand that this money has been contributed by a number of Medical Officers of the E. I. Co.'s Service in Bengal, (in sums averaging one gold mohur each), and that the object of the subscribers is to present to their quondam colleague, Mr. J. R. Martin, a handsome piece of plate, commemorative of their personal respect and obligations to him for his exertions to procure an improved scale of pensions.

I am desired by the Contributors to request that you will do them the favor to communicate these their wishes in suitable terms to Mr. Martin, and that you will farther be good enough (in concert with him), to select an appropriate piece of Plate, from the workshop of Messrs. Green and Ward, bearing the inscription adopted unanimously at the Meeting.

A corrected list of the Subscribers will be forwarded by the next mail. It is possible that there may be some few additions to their number in India, but we cannot doubt that those members of the Service now at home will take the opportunity of joining in the Testimonial, if they are made aware by any means of the steps taken here, and from these sources it is hoped the subscription may amount to £300. altogether.

I have the honor to be,
Gentlemen,
Your Obedient Servant,
(Signed) A. Garden,
*Chairman of Committee.*

*Calcutta, 7th January,* 1845.

---

*Calcutta, November* 13*th,* 1844.

The Martin Testimonial.

The Meeting of the Subscribers to the Martin Testimonial was held yesterday evening, in the Town Hall, when Dr. A. Garden was in the Chair. After the reading of a Report, by Dr. Duncan Stewart, the following Resolutions were unanimously adopted:—

Proposed by Dr. Jackson, and seconded by Dr. W. B. O'Shaughnessy, and resolved,

1st. That the Report is satisfactory, and be published in the daily papers.

Proposed by Dr. A. Smith, and seconded by Dr. Finch,

2nd. That it is the opinion of this Meeting, that the most appropriate form of Testimonial will be that of one massive piece of Plate, with a suitable inscription.

Proposed by Dr. Goodeve, and seconded by R. O'Shaughnessy, Esq.,

3rd, That the following inscription be thereon engraved:—" Presented to James Ranald Martin, Esquire, late of the E. I. Co.'s Bengal Medical Service, by his brother Officers, in token of their personal regard, and their obligations to him for his successful exertions in procuring for them the improved scale of pensions after 21 years' service."

Proposed by Dr. Jackson, and seconded by Mr. Egerton,

4th. That Colonel Young, Mr. John Turner, and Dr. A. R. Jackson be requested to see the above Resolution carried into effect (by Messrs. Green and Ward of London), in communication with Mr. Martin.

Proposed by Dr. D. Stewart, and seconded by Dr. Garden,

5th. That a complete list of contributors and of payments up to the present time be again published, and that the subscription book remain open till 1st January, 1845.

Proposed by Mr. Egerton, and seconded by Dr. Smith,

That the thanks of the Meeting are due to the Chairman.

---

To Colonel James Young, John Turner, Esq.,
and A. R. Jackson, Esq. M.D.

GENTLEMEN,

I have had the honor to receive, through you, the letter of Dr. Garden, Chairman of the Committee of Medical Officers of the Bengal Army, of 7th January, 1845, together with copies of resolutions adopted by my late brother officers at a Meeting held on the 13th November, 1844, at the Town Hall of Calcutta.

The third Resolution conveys to me, on their part, the expression of " their personal regard, and of their obligation to me for my successful exertions in procuring for them the improved scale of pensions, after twenty-one years' service:" and, by another resolution, I am presented with a " massive piece of plate," in token of the sense entertained by the subscribers of these, my exertions.

It is not possible for me, gentlemen, to express in adequate terms the feelings with which I receive this testimony of the esteem and good will of those officers—many of them my best friends—with whom I served during the better part of my life. I can only say, that I accept it as one of the most gratifying and honorable rewards that I could receive from any quarter; and that I shall ever esteem this expression of " the personal regard" of my brother officers as one of the most pleasing circumstances of my life.

In regard to the improved scale of pensions recently accorded to the Medical Departments of India, I may be permitted to say, that, owing to circumstances of a personal nature, I was enabled to secure access to persons in authority, and I was thus enabled to urge forward this important question. I did so during three years, on the score of its justice—on that of its substantive value—and with a view to set aside one of those relative distinctions which I believed to be injurious to the interests of the public service at large. That my exertions should prove acceptable to the Medical Department of the Bengal Army especially, would alone have been to me a sufficient reward, and I looked to none besides.

While I request that you will do me the favor to convey these my sentiments of esteem and grateful regard to Dr. Garden, and to the numerous body of my brother officers whom he represents, I beg, at the same time to offer to you, Gentlemen, the expression of my sincere thanks for the handsome and friendly terms in which you have been pleased personally to convey to me this most flattering testimonial.

I have the honor to be,
Gentlemen,
Your faithful and obedient Servant,
(Signed) J. R. Martin.

*Grosvenor Street, London, March* 12, 1845.

---

*London, March* 17th, 1845.

To Dr. A. Garden, Chairman to the "Martin Testimonial" Committee, Calcutta.

Sir,

Almost immediately on receiving your letter of instructions regarding the Martin Testimonial, and conveying a remittance of £250., one of our members, Col. Young, in order to save a mail, took upon himself to address you a separate letter, acknowledging receipt, and reporting progress, so far. In continuation of that letter, we have now the honor more formally to apprize you of our proceedings towards completing the execution of your Committee's instructions, and in furtherance of the noble design contemplated by the Medical Body of Bengal.

We accordingly have the pleasure now to enclose Mr. Martin's reply to our communication, which we doubt not will be gratifying to his "brother officers."

The Bill for £250. after presentation and acceptance, was handed over to Mr. Martin, who accompanied our colleague, Mr. Turner, to Messrs. Green and Ward's, and made choice of an appropriate and handsome piece of plate, on which the prescribed Inscription is now being engraved.

In reference to the concluding paragraph of your letter, in which you suggest that the Medical Officers now at home should be referred to, with a view to afford them an opportunity of contributing to this testimonial, we beg to state that we were about to take steps to that effect, when, at the personal request of Mr. Martin, we desisted, and have relinquished the intention altogether.

In a formal Report such as this, of proceedings in a public matter, we ought not, perhaps, to obtrude any mention of ourselves or our feelings; yet, we have all of us been so long acquainted with the eminent professional and public character of Mr. Martin, besides being personally and familiarly intimate with that excellent and amiable man, that we reall[illegible] feel indebted to your Committee for having selected us to perform th[illegible] pleasing office of communicating to our esteemed friend the gratifying an[illegible] merited compliment, voted to him by his late Colleagues.

We beg to subscribe ourselves, Sir,

Your obedient Servants,

(Signed) J. YOUNG,
JOHN TURNER,
A. R. JACKSON.

---

(From the *Bengal Hurkaru*, May 22nd, 1845.)

We have much pleasure in giving a place to Dr. Falconer's letter, by which we learn that the Members of the Medical Service in England, duly appreciating the disinterested exertions of Mr. J. R. Martin to obtain the boon for them, had expressed a desire to join in the subscription to the Martin Testimonial, and were only prevented by his request, that no such addition might be made—a request founded in a considerate regard for the circumstances of Medical Officers at home. We quite agree, however, in thinking that it is due to Dr. Falconer and his brethren of the Profession, that their good intentions and their movement in this matter should be generally made known, for their coming forward, as they have done, is highly creditable to them, while the delicacy of Mr. Martin's motive for desiring that they should desist from their purpose is not less honorable to him, and cannot fail to raise him, if that be possible, in the estimation of the Service, and of all to whom his conduct on this occasion becomes known. The Bengal Medical Officers at home have been enabled, however, to record their participation in the sentiments of esteem and gratitude towards Mr. Martin, which have been expressed by the faculty in this country:—

*Oriental Club, March* 29, 1845.

MY DEAR MARTIN,

I have received your note, which I have communicated to my Medical friends in the Service here, and I have conferre[illegible] with Turner on the subject.

As you have with such handsome consideration declined the addition o[illegible] our mite to the "Testimonial," it is not for us to press the matter upo[illegible] you; but I am glad that the occasion has furnished us with the oppor[illegible] tunity of assuring you that we entirely participate in the sentiments [illegible] gratitude and esteem towards you, for your earnest and disinterested e[illegible] ertions in the matter of the boon, professed by our brethren in the Servi[illegible] in India, and that we would have gladly united with them in contributi[illegible] to the Martin Testimonial, had not your own wishes been against it. W[illegible] were quite ignorant of what had passed between you and the Home Con[illegible] mittee on the subject, and should you have occasion to communicate wi[illegible] the Committee in India, we beg that you will mention what our intentio[illegible] were. With the offer of our best wishes for your welfare,

Believe me, my dear Martin,

Your's very sincerely,

H. FALCONER.

To J. R. Martin, Esq., 71 A, Grosvenor Street.

